# Exploring physicians’ decision-making in hospital readmission processes - a comparative case study

**DOI:** 10.1186/s12913-018-3538-3

**Published:** 2018-09-19

**Authors:** Malin Knutsen Glette, Tone Kringeland, Olav Røise, Siri Wiig

**Affiliations:** 1 Faculty of Health, Western Norway University of Applied Sciences, Haugesund, Norway; 2 Division of Orthopedic Surgery, Oslo University Hospital, Oslo, Norway; 3 Faculty of Health Sciences, SHARE – Centre for Resilience in Healthcare, University of Stavanger, Stavanger, Norway; 4 Institute of Clinical Medicine, University of Oslo, Oslo, Norway

**Keywords:** Hospital readmissions, Patient safety, Hospital discharge, Patient handovers, Decision-making

## Abstract

**Background:**

Hospital readmissions is an increasingly serious international problem, associated with higher risks of adverse events, especially in elderly patients. There can be many causes and influential factors leading to hospital readmissions, but they are often closely related, making hospital readmissions an overall complex area. In addition, a comprehensive coordination reform was introduced into the Norwegian healthcare system in 2012. The reform changed the premises for readmissions with economic incentives enhancing early transfer from secondary to primary care, making research on readmissions in the municipalities more urgent than ever. General practitioners (GPs) and nursing home physicians, have traditionally held a gatekeepers function in hospital readmissions from the municipal healthcare service, as they are the main decision-makers in questions of hospital readmissions. Still, the GPs’ gatekeeper function is an under-investigated area in hospital readmission research. The aim of the study was to increase knowledge about factors that lead to hospital readmissions among elderly in municipal healthcare, with special attention to GPs’ and nursing home physicians’ decision making.

**Method:**

The study was conducted as a comparative case study. Two municipalities affiliated with the same hospital, but with different readmission rates were recruited. Twenty GPs and nursing home physicians from each municipality were recruited and interviewed. Forty hours of observation were conducted during the huddles in one long-term and one short-term nursing home in each municipality.

**Results:**

Seven themes describing how different factors influence physicians’ decision-making in the hospital readmission process in two municipalities were identified. Poor communication, continuity and information flow account for hospital readmissions in both municipalities. Several factors, including nurse staffing and competence, patients and their families, time constraints and experience affected physicians’ decision-making.

**Conclusion:**

Communication, continuity and information flow contributed to hospital readmissions in both municipalities. The cross-case analysis revealed slight differences between municipalities. More research focusing on GPs’ and nursing home physicians’ decision-making, nursing home nurses and home care nurses’ experience of hospital readmissions and discharges is needed.

**Electronic supplementary material:**

The online version of this article (10.1186/s12913-018-3538-3) contains supplementary material, which is available to authorized users.

## Background

Readmissions 30 days after hospital discharge are considered an international problem with readmission rates ranging from 10 to 30% across borders [1–4]. Hospital readmissions are associated with higher risk of adverse events, especially in elderly patients [5, 6]. Furthermore, hospital readmissions are an economic burden for the healthcare system and consume healthcare resources [7, 8].

Readmissions often result from a combination of disparate factors [9, 10]. High readmission rates can be indicative of suboptimal patient treatment and/or an unnecessary use of resources. For patients with acute deterioration as a part of their clinical picture, however, a low threshold for readmissions can be indicative of a higher quality of care [9]. There is a higher prevalence of readmissions in patients with chronic illnesses or conditions that limit their ability to perform their activities of daily living. Other factors that may influence hospital readmissions are patient age, availability of social support and access to adequate care after hospital discharge [7, 11].

The number of readmissions in Norwegian hospitals has, in accordance with international numbers, increased the past years [12]. A national mapping of the prevalence of hospital readmission rates showed differences in readmission rates between municipalities linked to the same hospital, and with similarities in population and location in relation to the hospital [9]. Previous research on hospital readmissions does not explain these differences.

### The role of general practitioners and nursing home physicians in hospital readmissions

In a Norwegian context, general practitioners (GPs) provide medical care in the municipal healthcare services (GP offices, nursing homes and emergency rooms) and are responsible for making hospital referrals as needed, making them the gatekeepers to the secondary healthcare service [13, 14]. Consequently, GPs play a significant role in hospital readmissions [15, 16]. Research has shown that other factors in physicians’ decision-making -- other health personnel, colleagues, physicians’ personal factors, patients and their families -- can influence the decision-making process [15, 17]. Most of the research on this topic has been conducted outside of municipalities.

Studies aiming to map reasons for hospital readmissions have tended to take a patient-or hospital-centered focus that excludes the perspectives of GPs and nursing home physicians [7, 11, 18, 19].

### Case-based decision theory

The quality and costs of the healthcare services are based on healthcare professionals’ everyday decisions [17]. Medical decisions often include complex ethical problems involving numerous stakeholders (e.g., the patient, the family, physicians, physician colleagues) and decision outputs in form of an action such as a test, a treatment regimen or a hospital readmission.

Case-based decision theory (CBDT) suggests that people’s actions are based on their previous actions in similar past situations [20]. It can be useful in understanding how GPs and nursing home physicians make their decisions. The decision-maker takes factors, both personal experience and that of others, into his or her decisions. The similarities with other decision-makers’ previous problems and the attributes that they share will affect the extent to which they are influenced when making decisions [20]. In the medical context, CBDT assumes that GPs’ working experience will affect their decision-making. Consulting other physicians and reading patient journals will be two ways of collecting the experiences of other decision-makers.

### Purpose of the study

The purpose of this study was to increase knowledge of factors influencing hospital readmissions of elderly from a municipal healthcare perspective. We focused on how GPs and nursing home physicians make decisions about hospital readmissions. We wanted to investigate which and how different factors influence GPs’ and nursing home physicians’ decision-making in the hospital readmission process, and the contributions of other healthcare professionals.

Through qualitative interviews and observations of physicians’ decision-making, this study illustrated factors affecting hospital readmissions from a municipal perspective, thereby better enabling us to suggest future measures to reduce hospital readmissions among the elderly patient group.

## Methods

### Study design

**Fig. 1:**
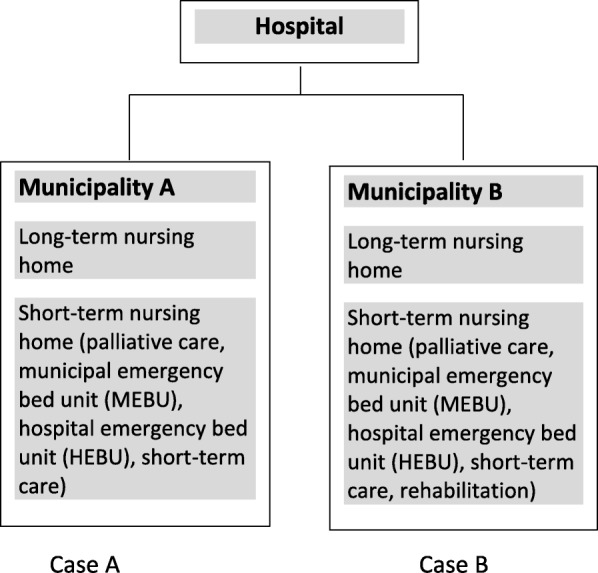
Overview of cases

This study was conducted as a contrasting comparative case study of two Norwegian municipalities. A case was defined as a municipality with affiliated primary healthcare services and the affiliated hospital. Two cases were included in the study (Fig. 1). The two municipalities were affiliated with the same hospital and were selected based on their different readmission rates at the time of recruitment (19.2% in Municipality A and 15.2% in Municipality B in 2014). As we assumed that these differences were robust, we decided to use the contrasting case design – anticipating contrasting results and a possible variation in factors influencing the decision-making process [21]. Further, the investigation of two cases allowed for comparison and the exploration of potential differences and similarities across and within cases [22]. The purpose was to recruit cases with contrasting rates but affiliated with the same hospital for a qualitative exploration, not to analyze the readmission rates over time.

### Sample and recruitment of municipalities

The municipalities were recruited based on results from the national quality indicator “30 day readmission after hospital stay” published by the Norwegian Institute of Public Health [9]. The quality indicator calculates the risk-adjusted likelihood for readmissions within 30 days of hospital discharge for elderly somatic patients (67 years and older) within 11 diagnosis groups where the total indicator is calculated and published every year [9]. The municipalities were similar in a Norwegian context in terms of similar population size and proximity to the hospital (Table 1).

**Table 1:** Demographic Overview of Cases

Description	Municipality A	Municipality B
Distance from the hospital	5–35 km	Hospital placed within the municipality
Inhabitants	Approximately 40,000 (rounded down)	Approximately 40,000 (rounded up)
Physician Full Time Equivalent (FTE) per 10,000 habitants	8	9
Degree of nursing home coverage or coverage in institutions for persons 80 years and older as a percentage of the corresponding age group in the population	11%	15%
Emergency room	1	1
Municipal Emergency Bed Unit (MEBU)/ Hospitals Emergency Bed Unit (HEBU) short-term nursing home/rehabilitation/palliative care	1	1
MEBU/HEBU distance to hospital	5 km from the hospital	Less than 5 km from the

(Numbers from municipal – state reporting (KOSTRA), 2016)

In each municipality, the head of the health department and the director of health provided the contact information of eligible informants. The researchers established contact by sending an information letter inviting all GPs and nursing home physicians in each of the two municipalities to participate. The first author made the second contact and scheduled the interviews. All informants had to work in either Municipality A or Municipality B. Municipal leaders collaborated in the recruitment of nursing homes. The first author met with the administrators of each nursing home to plan the research.

### Context

The Norwegian healthcare service is grounded on the welfare model and includes publicly funded health services, equal social rights and equal access to healthcare services [23]. The healthcare service consists of separate primary and secondary services funded separately by the municipalities and the state.

Each municipality provides healthcare services to its citizens at its own discretion, within certain regulations, leading to differences in how the healthcare service is delivered (e.g., resources, nursing home coverage, staffing level and staff competence) in nursing homes [24]. The organization of the healthcare service is similar in the two municipalities considered here. Emergency room (ER) shifts are staffed by GPs with emergency room duties, but also interns, doctor temps and physicians working in small permanent positions. The two municipalities had similar institutional coverage (nursing homes) for persons 80 years and older (percentage of the corresponding age group in the population) (Table 1).

As coordination of healthcare professionals is essential in providing safe, high-quality healthcare [23] all municipalities are legally required to sign agreements of cooperation between the primary and the secondary healthcare services. This includes agreements on hospital admissions, hospital discharge and rehabilitation, to provide holistic healthcare service [24].

The Norwegian healthcare system underwent comprehensive changes following the implementation of the Coordination Reform in 2012 [25], that was designed to develop a more holistic healthcare service and better collaboration among the healthcare services. The result has been shorter hospital stays, heavier patient flow and greater pressure on the primary healthcare services, mainly due to economic incentives favoring short hospital stays. The municipalities must now pay the hospital costs of keeping a patient who is determined not to need further hospital care [9, 12, 26]. In addition, the coordination reform has resulted in the establishment of municipal emergency bed units (MEBUs) for patients that the municipality itself can examine, treat or provide care for. These units are mainly for patients with worsening illnesses, where treatment has already been planned, and whose condition does not require hospitalization [27]. In the current municipalities, the MEBUs are staffed with nursing home physicians during regular business hours, and by ER doctors on evenings, nights and weekends. The MEBUs are located in the short-term nursing homes included in this study. GPs, nursing home physicians, and ER doctors can refer patients to MEBUs after diagnosing them and developing a treatment plan [28].

### Data collection

We used semi-structured interviews and observations to collect data for this study.

Twenty participants were recruited to take part in the study: eight GPs, one nursing home physician and one physician working part-time in the ER and part-time in the nursing home in each municipality. The participants differed in their years of experience (Table 2) and medical specialties (e.g., geriatric competence, general practice, emergency medicine, psychiatry). Some of the participants had not had an internship, which is now required of all graduates, but were working as physicians with all rights and responsibilities.

**Table 2:** Distribution of physicians’ years of experience

Years of experience	Included physicians (municipality A)	Included physicians (municipality B)
0–5	5	1
5–10	2	3
10–15	2	2
< 15	1	4
Mean years of experience	9,6 years	15 years

The first author conducted the interviews from September 2016 to February 2017. Each interview lasted for approximately 30 min. The interview guide (Additional file 1) was developed using case based decision theory and Systems Engineering Initiative for Patient Safety (SEIPS) model [29] and consisted of following topics: hospital admissions based on medical justifiability; external influences (e.g., patients, next of kin, other health care professionals): personal factors (e.g., experience, fear of consequences, personal relations/feelings). Our conceptualization of readmission was based on the definition of the quality indicator criteria (readmissions among elderly somatic patients - > 67 years). However, no constraints were put on the interviewees in regard to certain types of hospital readmissions (diagnose specific hospital readmissions). The physicians were encouraged to talk about hospital readmissions in general to secure the richest possible data material on the factors affecting their decision-making in hospital readmissions. The interviews were recorded and later transcribed.

The first author conducted observations in the huddle of one long-term and one short-term nursing home in each municipality from December 2016 to June 2017. This resulted in approximately 40 h of observation. The four physicians leading the huddles during the observations were also interviewed. Observations and interviews were conducted at separate times to secure that the interaction data would be captured without any researcher involvement [30]. The purpose of the observation material was to fill in or support the interview material and strengthen the validity of the data material. The huddle was selected because it involves decision-making, cooperation between physician and nurses and the participation of patients and next of kin in medical decisions. An observation guide (Additional file 2) was developed based on the following themes: interaction between GPs/nursing home physicians, physicians’ colleagues and other health personnel in questions of hospital readmissions, interaction between GP/nursing home physician and patients and family, and the readmission process. Observation notes were taken throughout the observations.

### Analysis of interviews and observation data

All interviews and observations were transcribed and analyzed according to UH Graneheim and B Lundman [31] content analysis to map factors that can affect hospital readmissions.

The GPs’ and nursing home physicians’ experience of hospital readmissions was extracted from the interview material (the unit of analysis) in the form of meaning units. The meaning units were then condensed, coded and organized under categories and subcategories as shown in Table 3. Seven themes in each municipality describing factors that influence physicians’ decision-making in hospital readmissions emerged.

**Table 3:** Content analysis municipality B, Theme 2

Theme	Category	Sub-category	Codes
T2: Lack of coordination, access to and continuity in the patient information flow	Information exchange	Lack of coordination between primary and secondary healthcare services	*Communication between the municipal healthcare service and the hospital during hospital discharge, is not good enough*
		Inadequate access to patient information	*Lack of adequate information exchange within the municipal healthcare service, and between the hospital and the municipal healthcare service*
			*Medication- lists which are not up-to-date leads to additional work for the receiving physician*
			*Status on resuscitation is not always clarified*
			*Physicians baser their decisions on clinical assessments, the patients general condition and results from available measurements*
	Continuity	Lack of continuity in the patient treatment	*It is difficult to know about previous hospital admissions; if the patients’ medical problem is already known and how the patients coped after ended shift at the emergency room*
			*Hospital admissions can become necessary because the nursing home physician don’t have the opportunity to do follow-ups during weekends and evenings at MEBUs*

The observation material was read through several times to arrive at a sense of the whole and divided into meaning units which were condensed. The underlying meaning of the condensed units was interpreted and divided into themes and subthemes, resulting in two themes in Municipality A and three themes in Municipality B [31].

The themes of the interview material were structurally introduced, and the themes of the observations were used to substantiate the results of the interview material.

The cases were first analyzed individually to identify factors affecting GPs’ and nursing home physicians decision-making. Second, a cross-case analysis was conducted across municipalities to map differences, similarities and patterns [22].

## Results

### Theme 1: Transference of responsibility from the hospital to the municipal healthcare service

GPs from both municipalities described a pronounced shifting of medical responsibility to the municipal healthcare service. Several GPs stated that patients had been discharged from the hospital with unresolved medical issues or incomplete treatment.

The problem is, they [the hospital doctors] exclude a bone fracture, but they don’t investigate any further why the patient had fallen in the first place. (GP, Municipality B).

It was clear that premature hospital discharge after completed intravenous (IV) antibiotic treatment was a factor in hospital readmissions in both municipalities. Patients’ medications were often changed from IV to oral antibiotic treatment on the day of discharge. The effect of the change in medication had therefore not been observed and the patients deteriorated, requiring readmission to resume IV treatment.

The patients were described as complex and in need of advanced treatment. Some of the GPs and nursing home physicians reported that they had to take on responsibilities for continued examinations, tests or referrals to other fields of expertise. Moreover, GPs in both municipalities had been urged to keep the patients in the municipal healthcare service when they wanted to refer the patient to the hospital.

We’ve received phone calls from the head physician at the hospital, explaining to us that the hospital is full and that we should re-hold all hospital admissions. But… this can be compared with them saying that we sometimes admit patients to the hospital for the fun of it… if you know what I mean. (GP, Municipality A).

In Municipality A, some GPs and nursing home physicians reported that the hospital was trying to stop hospital admissions from nursing homes, on the grounds that elderly patients with multiple morbidities were less treatable. Some of the interviewees stated that the hospital physicians did not know which treatments could be executed at the nursing homes. Patients were, for example, discharged from the hospital back to the nursing home with orders for tests, such as daily liver blood samples, that could not be run at the nursing home. This resulted in a hospital readmission for the required care.

In Municipality B, some GPs claimed that the hospital encouraged use of MEBUs when the GP referred a patient to the hospital. Some GPs also reported that the hospital physicians sometimes misused the MEBUs by asking the GP to admit the patient to a MEBU without the GP having the time or capacity to ascertain if this patient was such a candidate or to plan a course of treatment.

### Theme 2: Lack of coordination, access and continuity in the patient information flow

Physicians in both municipalities complained about a lack of cooperation between the municipal healthcare service and the hospital during the discharge process. In Municipality A, there were questions about the criteria used to determine when a patient was ready for discharge. Further, the GPs wished for a better cooperation during the discharge process. Incidents like discharging a complicated patient on a Friday afternoon, knowing the limited nursing home resources at this time, was described as one problem. Some physicians found it unsettling to be excluded from medical discussions of patients that they had been treating for years. One physician described it as working in two different worlds*,* and stated that he missed being a part of a greater team around the patient.

I do believe, that in the relay race, when you pass the baton to the next sprinter, it should be a smooth transfer, not “here comes the stick,” you know, “catch it if you can!” (GP, Municipality B).

In Municipality B there were vigorous complaints about insufficient information exchange between healthcare institutions, such as the hospital, nursing homes and GPs. There was incomplete written information after stays at MEBUs and incomplete written information when the GP referred patients to MEBUs. Hospital stay summaries (HSS) were in many cases unsatisfactory and often received too long after the hospital stay, making it difficult to make informed decisions. Both municipalities reported this problem. The patients’ medication summary was also often sent late and without sufficient description of changes of medication or indication for these changes. The nursing home observations showed several examples of the nursing home physicians not having access to necessary information about newly admitted patients (to MEBU), and needed to call the hospital to have the information transferred to the nursing home.

GPs working at the ER described not having access to the HSS or to patients’ medical records. This could lead to difficulties, as most patients could not recall what examinations or assessments had been done at the hospital. Not having this information was cited as a reason for readmission, especially if the ER was busy.

We have to call the on-call physician (to get information) because the patients don’t have the hospital stay summary along with them, and they don’t remember what have been done and said at the hospital. And they’re like “I got one pink pill and two green pills” and then I have to call, and that is annoying, especially if it is busy. (GP, Municipality B).

She had a known heart condition and a GP would probably have handled it differently. But as an emergency room physician without information about the patient, a hospital admission was the only solution. (GP, Municipality A).

Furthermore, the observation material showed limited continuity in the nursing homes due to low physician coverage, especially in the long-term nursing home of municipality A. Some physicians had arrangements with the nurses to be available by telephone in the afternoons and on weekends in the hope of preventing unnecessary hospital admissions.

If they can’t get hold of me, and the emergency room doctor has to come, it can be an intern or a physician without nursing home experience. And he sees, you know, a blood pressure at 60 and a CRP counting over 100, and… they’ll admit the patient to the hospital. (GP, Municipality A).

Another popular topic in both municipalities was clarification of do not resuscitate-status (DRN), which is used to determine if a patient in cardiac arrest should receive cardiopulmonary resuscitation or not. There was a consensus on the importance of clarifying DRN-status in nursing home patients with multiple morbidities and to clarify how much invasive treatment an acute patient should receive. This clarification was meant to spare the patient unnecessary suffering and to make it easier for physicians who are unfamiliar with the patient (as in the ER) to make treatment decisions. DRN-status and a planned course of treatment was a very important factor in questions of hospital readmissions, but such an assessment had not always been made.

### Theme 3: High workload and time pressure increase chances of readmission

Physicians in both municipalities claimed that when there was a heavy workload combined with limited time, it was easier to admit the patient to the hospital than to find an alternative. This was especially a problem in the ER. Admitting patients to MEBUs required a diagnosis and a treatment plan, so hospitalization was faster. Looking up missing information about the patient was also time consuming, and a busy ER could result in a hospital admission.

In Municipality B, some GPs were concerned with a lack of nursing home placements. They found it hard to place the patients who needed it. They argued that the hospital had a greater impact when assignments of nursing home placements were being made, so admitting the patient to the hospital could help to secure the patient a bed in a nursing home.

### Theme 4: The importance of patient and the family preferences

GPs and nursing home physicians in both municipalities insisted that the patients’ preferences and wishes were important factors in their decision-making. The patients’ wishes were not always medically justifiable, so although they were always given serious consideration, they could not always be respected. In addition, the patients were seen as sources of important information.

Seventy to 80% of our diagnostics is based on a comprehensive medical history, an anamnesis, so we have to listen to the patients! (GP, Municipality A).

The patients’ family was another source of information. The family spoke on behalf of patients who were unable to speak for themselves. There was one example of the physician – next-of-kin relationship in the observation material, where the nursing home physician urged a patient’s husband to monitor his wife for side effects of a new medication. Some physicians viewed next of kin as exerting pressure on medical decisions, for example in regard to hospital readmissions.

The family wanted the patient to be admitted to the hospital no matter what. I believed that the patient was dying and wanted to give him palliative care at the nursing home. But after extreme pressure from the patient’s family, and with me as a novice physician not being confident enough to say “no, he cannot go to the hospital,” the patient was placed in an ambulance and passed away during transportation. (GP, Municipality A).

The observation material in one long-term nursing home revealed how a patient’s family could demand hospital examinations that the nursing home physician had refused as medically unnecessary. In one example, a patient’s family wanted to prolong treatment. The patient’s own wishes were unclear, and the GP’s and nurses’ assessments of the patient’s best interests were inconclusive. In this case, the family’s wishes took priority.

If there were disagreements between the patient and the family, the patient’s wishes came first. As much as his or her condition allowed, the patient was encouraged to participate in the decision process.

If the patient’s family’s demands are unreasonable, and they wanted us to do unnecessary examinations which could be a burden for the patient, then I wouldn’t admit the patient to the hospital on those terms. (GP, Municipality B)

Lastly, social factors such as the patient living alone, not having a social network or a family, could be a reason for readmitting the patient to the hospital for the sake of the patient’s care and wellbeing. If available, in such a situation, the MEBUs could be an option.

### Theme 5: The nurses are the physicians’ extended ears and eyes

In both municipalities there was an agreement that nurses were an important source of information. Especially in the nursing homes, nurses were described as the GPs’ eyes and ears. The nurses made valuable observations and gathered important information to make medical decisions. Sometimes the physicians’ decisions were largely based upon the nurses’ reports. Observations from the long-term nursing homes showed that the nurses were organizers, patient ambassadors, and information sources by monitoring the patients, keeping written records and making oral reports during huddles.

The nurses can do closer observations than the physicians can do. That is to say, they have a visual observational foundation and a symptomatic observational foundation which is better than ours maybe … or more detailed … (GP, municipality A).

There were several examples in the observation material of the nurses’ importance as information source. They reported changes in the patients’ condition, acute or over time (e.g. abnormal breathing, abnormal blood glucoses, rashes, abdominal pain), they reported effects or side effects of medication (need for medication changes, increased dosage or continued treatment) during the huddles. They informed the physician of reports that needed to be written, next of kin who wanted meetings and reminded them of planned examinations. At the same time, in both municipalities, nurses - especially nursing temps and night nurses - did not always have sufficient knowledge of the patients and their history. It was for example observed during a huddle, that a nurse could not answer a physician’s questions about a patient’s condition or the patient’s history as she was not familiar with that ward. The physicians described it as particularly difficult to get accurate information when they were doing their ER duties.

They [the nurses] are not necessarily familiar with the normal function level of the patient (…) I get plenty of telephones at the emergency room where they are telling me that the patient is ill, but they haven’t measured the blood pressure, not pulse, they don’t know anything else”. (GP, Municipality B).

The physicians stated that the nurses were highly competent and that their competence had improved over the years. At the same time, variation in the nurses’ competence was described. If the nurses were not able to perform a necessary procedure on a patient, a hospital readmission could be required.

If the nurses are very insecure, and if the tasks are too difficult, it can be a reason for hospital readmissions in my opinion. (GP, municipality B).

The physicians also noted that there sometimes was a discrepancy between the needs of the patient and the resources of the nursing home. For example, not all nursing homes were, at all times, capable of offering round-the-clock care to a patient who needed constant supervision. Nurses often expressed these concerns, and the physician would decide whether or not to admit the patient to the hospital based on those concerns.

In Municipality A there were reports of staff shortages in nursing homes, especially a lack of nurses at night and on weekends. Not having enough nurses on each shift caused problems when patients needed specialized nursing care, such as administration of antibiotics or morphine.

It is a problem when the patient gets ill during an evening shift and you know he needs supervision during the night [when a nurse is not working]. Then it is tempting to admit the patient to the hospital, because I know there are no nurses on call. But this is not a reason for a hospital admission. I can’t tell the on-call hospital physician I am admitting because there is no nurse here. But I believe it is dangerous, it is a dangerous practice to not have a nurse working at all times. (Physician, Municipality A).

### Theme 6: The patients’ safety comes first

GPs in both municipalities said that if patients had unresolved medical issues, were too unstable to stay at home, or if the patient’s needs outstripped the nursing home’s resources, a hospital admission would ensure the patient’s safety and reduce the risks of adverse events. Simultaneously, there was a great attention to the drawbacks of hospitalizing elderly, cognitively impaired patients. GPs with nursing home duties in both municipalities did their utmost to avoid admitting such patients.

Another way to ensure the patients’ safety was to consult with other physicians. Most physicians used hospital physicians to discuss treatment options, get advice and support for difficult decisions. Extensive use of conferring with hospital physicians to discuss matters or get advice, was noted during the nursing home observations, especially in the MEBUs.

### Theme 7: Experience has a bearing on physicians’ decisions

In both municipalities, novice GPs and those with more than two decades of experience agreed that experience affected their decision-making about hospital readmissions. The physicians stated that experience gave them a sharper clinical eye and made them more confident in their decisions. Moreover, experience offered awareness of personal limitations and a sense of knowing when to involve others in the patient treatment. Factors like unfamiliarity with the healthcare system and how it works, not knowing other options to hospital admissions and not knowing who to consult, were, by the included physicians, associated with inexperienced GPs or inconsistent GP coverage. Physicians in Municipality A reported that frequent GP turnover had been a problem.

## Discussion

In the following, we discuss the results according to previous research and the Case Based Decision Theory [20].

### Hospital readmissions

One of the purposes of the coordination reform was to decrease the demand for secondary healthcare services by enabling the municipalities to take on more of the secondary healthcare service’s tasks [32]. It has been documented that the reform has had this effect in the form of early hospital discharges and more complicated patients being discharged to nursing homes and home care services [33]. According to Kristoffersen and Colleagues [34] this is a natural consequence of the municipalities assuming more responsibilities. Despite this natural change in the patient group, the physicians in our study found that patients were being prematurely discharged. A reason for a too early hospital discharge can be explained by shift from a patient-centered to an economic perspective as a consequence of the Coordination reform [35]. Another explanation can be under-dimensioned capacity in the hospitals, forcing hospital physicians to discharge patients early, to open up beds for new patients or to stop hospital admissions due to high occupancy [36]. Lastly, hospital physicians can be trying to prevent unnecessary hospital readmissions. For example, hospital readmissions based on time constraints in the ER, GPs admitting patients to protect themselves (if inexperienced or insecure), cultural differences over when to admit or not, lack of knowledge of when to admit or not, or lack of agreement over which patients should be admitted [37]. There is a need to investigate the hospital physicians’ understanding of these matters to fully understand the factors that lead to hospital readmissions.

Our findings showing lack of coordination and continuity, and poor information flow between health service levels are supported by a recent report by Office of the Auditor General of Norway [38]. Much of the communication between the municipal healthcare service and the hospital is exchanged through written referral letters from GPs/nursing home physicians and through hospital stay summaries (HSS). Studies and reports have shown that HSS are sometimes received late [39], are of poor quality in descriptions of medical history, symptoms, medication and social network, and lack accurate documentation of test results [40, 41]. These challenges, which was also described in our results, could indicate that an improvement in written communication and better communication tools among health care services could reduce hospital readmissions. Still, the hospital discharge process is often executed under time pressure, leading to lack of time to provide proper HSS and adequate information to the receiving healthcare agency [42].

Inadequate communication and cooperation in planning of hospital discharges, have also been described by Holen-Rabbersvik et al. [43]. Such problems may result in hospital readmissions if the municipalities are not prepared to receive the discharged patients due to facilities, lack of competence or staffing, equipment or lack of institutional space [44]. Nevertheless, research has shown improved communication between the healthcare agencies in the wake of the Coordination reform, contradicting our results [45]. This can be indicative of differences between municipalities in coping with the new demands of the coordination reform. Such difference may also be reflected in readmission rates. Municipality A experienced a peak in their readmission rates shortly after the introduction of the Coordination reform (15.2% in 2009, 19.2% in 2014, 16.6% in 2015 and 15.9% in 2016) while municipality B remained stable (15,6% in 2009, 15.2% in 2014, 16.4% in 2015 and 15.9% in 2016).

Hospital physicians lacked knowledge of what treatment options were available in the municipal healthcare services. Research supporting these results shows that the hospital physicians do not always understand the role and function of the municipal healthcare service (31) and that GPs and hospital physicians have limited knowledge of challenges in hospital admission and discharge processes [46]. Better shared knowledge of the conditions under which the different health agencies are working could facilitate cooperation and coordination between the hospital and the municipalities.

Patient handover is a critical component of quality in care and patient safety [47]. An inconsistency in physician coverage, as shown in our study, can result in more patient handovers, increasing the risk for adverse events grounded in interruptions in the flow of information [48]. Then, the physicians’ access to information depends more on nurses’ ability to provide accurate and complete information, thus placing additional responsibility on the nursing staff.

### Factors affecting physicians’ decision-making

#### The patient and the family

As enshrined in the Norwegian Patient and Users Rights Act § 3–1 [49], the patient has the right to contribute to decisions pertaining to his or her health and care services. Our results confirm the findings of other studies, showing that both the patient and the family are influential in physicians’ decision-making [50–53]. Like McDermot and colleagues [51] we found that the patient’s wishes were a key factor in the physician’s decision-making. Simultaneously, both the patient and the family could pressure the physician into conducting medical exams or admitting the patient to the hospital.

#### The nurses

The nurses were described as an important source of information, and their competence in caring for the patient and in conveying adequate information affected physicians’ decisions. As in our study, variation in nurse competence in Norwegian nursing homes has previously been described by Bing- Jonsson and colleagues [54]. However, the competence was perceived as improving over time in the included municipalities. This improvement could be explained by new and more demanding requirements on municipalities under the Coordination reform, where failure to meet the new demands presumably could lead to more hospital readmissions.

#### Experience

The physicians’ experience varied in the two municipalities (Table 2.) Experienced physicians who are more confident in their decisions have in the literature been described to be less likely to admit patients to the hospital [51, 52]. This can imply that inexperienced GPs are less capable of resisting unreasonable demands from patients and next of kin, resulting in unnecessary hospital readmissions. Case-based decision theory (CBDT) suggests that all decisions are grounded in previous experience in similar past cases [20]. This could imply that inexperienced physicians who do not have a basis of comparison, will, when unfamiliar with the patient, admit the patient to the hospital.

#### Alternatives to hospital readmissions

MEBUs are a novelty in the Norwegian healthcare system. Similar to our study, a report on MEBUs [55] showed that many physicians did not have enough knowledge about the application of the beds and which treatment level the MEBUs were in and continued to admit eligible MEBU candidates to the hospital. The report further supports our results which showed that admitting patients to MEBUs required considerable paperwork, and was therefore perceived as time consuming [55]. A simpler system could decrease hospital admissions for MEBU candidates.

Recent research echoes our study and shows that there is a higher threshold to get nursing home placements or homecare services after the introduction of Coordination reform [56]. Combined with older and sicker patients being discharged, it is likely that lower nursing home coverage could affect readmission rates. In their investigation of resource utilization and quality in the healthcare service, the office of the Auditor General of Norway found little or no increase in nursing home capacity after the introduction of the Coordination reform [57]. Meanwhile the number of patients in homecare has increased, the patients are sicker, and have a greater need for care than before [33]. The nurses in homecare are also meeting new challenges, and the GPs and ER doctors are facing a new patient population. This study has not investigated the home care service, but more research in this area is necessary to understand hospital readmissions in totality. Still, these findings reveal several human, technological, and organizational factors e.g. Carayon et al. [58] influencing hospital readmissions, and can be useful in the quality and safety work and in reduction of hospital readmissions.

### Limitations of the study

The methodological limitations of case study research and qualitative research were under constant evaluation throughout the research period. Some confounders still need to be addressed when interpreting the results of the study. The cases of this study were selected through convenience sampling, meaning that the study could be vulnerable to selection bias. However, the study aimed to investigate two contrasting municipalities based on readmission rates at the time of recruitment, making convenience sampling appropriate. During data collection, publications of new readmission rates showed an equalization between the municipalities. This could be considered a limitation. The cases were, however, initially chosen based on contrasting rates to explore possible variations in the decision-making involved in readmission processes. Moreover, hospital readmissions were reported as a national and local problem, and there was a need for more knowledge about why differences between municipalities occur. Hence, the aim of learning more about what factors influence GPs’ and nursing home physicians’ decision-making in hospital readmissions in two municipalities affiliated with the same hospital was still relevant and followed throughout the research.

Further, it was difficult to recruit GPs and nursing home physicians because of their time constraints, and this limited our selection options. The convenience sampling and the limited selection options could have caused us to miss more experienced GPs in Municipality A, distorting our picture of the differences in GP experience between the municipalities. During the interviews it was sometimes difficult for the GPs to distinguish hospital readmissions from hospital admissions, and necessary from unnecessary readmissions as their experience with these cases were retrospective. More extensive observations could have given more accurate data on specific types of readmissions. Lastly, some of the GPs came from outside of Norway, so there were some language difficulties when transcribing the recordings. Still, we believe that we have captured all crucial information.

### Implications for practice and further research

The findings in this study reveals several factors that influence hospital readmissions, and can be useful in the quality and safety work and in reduction of hospital readmissions. Further, the findings give an understanding of the challenges facing physicians in the municipal healthcare service when making medical decisions. In accordance with our interpretation of CBDT, inexperienced physicians will more often be insecure in patient treatment, and could find it safer to admit the patient to the hospital sooner than experienced physicians would. This could indicate a need for a municipal support system for inexperienced physicians, since they make most decisions without support from their more experienced colleagues. To obtain insight into hospital readmissions, there is a need for more research in municipalities, especially on GPs’ and nursing home physicians’ decision-making, and nursing home nurses’ and home care nurses’ experience of hospital readmissions and discharges. Also noteworthy are municipal leaders’ experience of hospital readmissions and hospital physicians’ view of discharging patients to the municipalities.

## Conclusion

Lack of communication, inadequate continuity and poor information flow were problems causing hospital readmissions in both municipalities. Several other factors such as nurse staffing and competence, the patient and his or her family, time, and experience also affected the physicians’ decisions pertaining to hospital readmissions. The cross-case analysis showed only small differences between the two municipalities. 

## Additional files

Additional file 1: Interview guide GPs/nursing home physicians (DOCX 16 kb)

Additional file 2: Observation guide, physicians in nursing homes (DOCX 13 kb)

## Abbreviations

CBDT: Cased-based decision theory
ER: Emergency room
GP: General practitioner
HEBU: Hospital emergency bed unit
HSS: Hospital stay summary
IV: Intravenous
MEBU: Municipal emergency bed unit
