# Supplementary material for: Exploring physicians’ decision-making in hospital readmission processes - a comparative case study
Source: BMC Health Serv Res. 2018 Sep 19;18:725. doi: 10.1186/s12913-018-3538-3 (PMC6146774; doi:10.1186/s12913-018-3538-3)
Supplement: Supplementary file 1 — Interview guide GPs/nursing home physicians (DOCX 16 kb) [file 12913_2018_3538_MOESM1_ESM.docx]

# Interview guide – GPs/ nursing home physicians

**Introduction**

- Purpose of the study
- The use and storage of data
- Anonymity
- Duration and structure
- Consent to record the interview

**Background information**

- Work experience
- Years of experience as physician
- Years working in current position

**Hospital readmissions based on medical justifiability**

1. What do you experience as the most common reasons for you to choose to readmit a patient to the hospital?
2. When you are unsure if a hospital readmission is the right choice, what do you do?
3. What information do you need to make a decision about a hospital readmission? Do you have (easy) access to it?

**Hospital readmissions based on external influence**

1. What other reasons, than the patients’ medical condition, can be of meaning in questions of hospital readmissions?
2. Is decisions about hospital readmissions exclusively made by you, or is there others affecting this decision, if so, who and in what way?

| **Follow-up questions:**  What influence does the patient have in decisions of hospital readmissions?  What influence do the patient’s next of kins have in decisions of hospital readmissions?  How is colleagues (in yours or other institutions) influencing decisions of hospital readmissions?  How do other health personnel (such as nurses, assistant nurses, ambulance workers) influence decisions of hospital readmissions |
| --- |

1. What information do you need from other health personnel when you are making a decision about a hospital readmission? Is this information (easy) accessible?
2. To what extent do you mean that staffing levels and competence in the municipal healthcare service (nursing homes) affect decisions about hospital readmissions?

| **Follow-up questions:**  How do staffing levels and competence in the municipal healthcare service (nursing homes) influence decisions about hospital readmissions? |
| --- |

1. How are other health personnel supporting you in decisions of hospital readmissions?

**Hospital readmissions based on personal factors**

1. How do you believe that your work experience affects your decision about readmitting a patient?

| **Follow-up questions:**  Do you experience differences between experienced and inexperienced physicians? In what way? |
| --- |

1. Can you describe some factors in the assessment of a patient (related to hospital readmissions) which makes a decision difficult to make?
2. What role do patients’ wishes play in your decisions about hospital readmissions?
3. How do you involve patients and next of kin in decisions of hospital readmissions?
4. In the aftermath of a hospital readmission, have you ever thought that the readmissions was unnecessary? If so, what was the reason?
5. In the aftermath of not readmitting a patient, have you ever though that you were too reticent? If so, what was the reason?

**Hospital readmissions based on organizational factors – the Coordination reform**

1. Have you experienced any changes in the hospital readmission process in relation to the Coordination reform?

| **Follow-up questions:**  Have you noticed any changes in terms of need for hospital readmissions in relation to the Coordination reform? |
| --- |

1. What significance do you think early hospital discharges have on hospital readmissions?
2. Do you have any opinion as to whether the number of readmissions has increased after the introduction of the Coordination reform?
3. There are differences in readmission rates between municipalities who are linked to the same hospital. Based on your experience, what do you think can be the reason for this?

| **Follow-up question:**  Do you have any examples?  Is there any measures you believe could reduce hospital readmissions? |
| --- |

**Summary**

1. Is there anything you believe to be relevant to the topic, which has not been addressed in this interview?

- Review of the most important topics appearing during the interview.
- Clarify any potential misunderstandings.
- Additional comments?
