# Supplementary material for: Exploring physicians’ decision-making in hospital readmission processes - a comparative case study
Source: BMC Health Serv Res. 2018 Sep 19;18:725. doi: 10.1186/s12913-018-3538-3 (PMC6146774; doi:10.1186/s12913-018-3538-3)
Supplement: Supplementary file 2 — Observation guide, physicians in nursing homes (DOCX 13 kb) [file 12913_2018_3538_MOESM2_ESM.docx]

# Observation guide – Physicians in nursing homes

**Introduction**

- Aim of the study
- The use and storage of data
- Anonymity and confidentiality
- Consent to participate in the huddle

Fields note are written continuously.

**Coordination/interaction between nursing home physician, other physician colleagues and other health personnel in relation to hospital readmissions.**

- What role do physician colleagues play in decisions of hospital readmissions? How large is their part?
- What role do other health personnel such as nurses and assistant nurses play in decisions of hospital readmissions? How large is their part?
- How do the nursing home physician interact with his or hers colleagues (physician colleagues and other health personnel) in questions of hospital readmissions.
- How do physician colleagues and other health personnel influence the physician’s decisions in questions of hospital readmissions?
- What information is requested from patients/next of kin/ physician colleagues/other health personnel in questions of hospital readmissions? How is this information used?
- What technological tools are used in the information exchange and in the coordination between the different healthcare services in questions of possible hospital readmissions? In what way?

**Coordination between nursing home physician/ GP and patient/ next of kin**

- How do the physician cooperate with the patient/next of kin in questions of hospital readmissions?
- How do the patient/next of kin influence decisions about hospital readmissions?

**The readmission process**

- How does the actual readmission process take place?
- Is the readmission process equal every time?
- Is the readmission process different between physicians?
- What adjustments are made between patients with different needs?
- Is there any reasons for hospital readmissions than purely medical reasons?

**Summary**

- Member check
- Clarify any potential misunderstandings
- Additional comments.
